# Recognition of lettuce downy mildew effector BLR38 in *Lactuca serriola* LS102 requires two unlinked loci

**DOI:** 10.1111/mpp.12751

**Published:** 2018-11-06

**Authors:** Alexandra J. E. Pelgrom, Jelle Eikelhof, Joyce Elberse, Claudia‐Nicole Meisrimler, Rob Raedts, Joël Klein, Guido Van den Ackerveken

**Affiliations:** ^1^ Plant–Microbe Interactions, Department of Biology Utrecht University Padualaan 8, 3584 CH, Utrecht the Netherlands; ^2^ BASF Vegetable Seeds PO Box 4005, 6080 AA, Haelen the Netherlands

**Keywords:** *Bremia lactucae*, downy mildew, effector, gene dosage, hypersensitive response, *Lactuca sativa* (lettuce), resistance

## Abstract

Plant‐pathogenic oomycetes secrete effector proteins to suppress host immune responses. Resistance proteins may recognize effectors and activate immunity, which is often associated with a hypersensitive response (HR). Transient expression of effectors in plant germplasm and screening for HR has proven to be a powerful tool in the identification of new resistance genes. In this study, 14 effectors from the lettuce downy mildew *Bremia lactucae* race Bl:24 were screened for HR induction in over 150 lettuce accessions. Three effectors—BLN06, BLR38 and BLR40—were recognized in specific lettuce lines. The recognition of effector BLR38 in *Lactuca serriola *LS102 did not co‐segregate with resistance against race Bl:24, but was linked to resistance against multiple other *B. lactucae* races. Two unlinked loci are both required for effector recognition and are located near known major resistance clusters. Gene dosage affects the intensity of the BLR38‐triggered HR, but is of minor importance for disease resistance.

## Introduction

Plants rely on their innate immune system to fend off a wide range of pathogens. Initial detection of pathogens occurs through the recognition of conserved features, collectively referred to as pathogen‐associated molecular patterns (PAMPs), by specialized receptors on the plant cell surface and the subsequent activation of pattern‐triggered immunity (PTI). Plant pathogens can evade or suppress PTI by secreting effector molecules that act in the plant apoplast or intracellularly, thereby inducing a state of effector‐triggered susceptibility. However, resistant host genotypes can recognize host‐translocated effectors by intracellular nucleotide‐binding and leucine‐rich repeat receptors (NLRs) and launch an effector‐triggered immune response that is frequently associated with a hypersensitive response (HR), a form of localized cell death (Jones and Dangl, 2006).

Two classes of host‐translocated effectors have been discovered in plant‐pathogenic *Phytophthora* and downy mildews: the Crinkler (CRN) effectors and RXLR(‐like) effectors (Anderson *et al*., 2015). CRNs are modular proteins, most of which have an N‐terminal signal peptide, followed by LXLFLAK and DWL domains. The analysis of 315 *Phytophthora* CRNs resulted in the identification of 36 conserved C‐terminal domains (Haas *et al*., 2009). The N‐terminus of RXLR‐like effectors is characterized by a signal peptide, followed by an RXLR or related motif, such as GKLR or QXLR, in the first 40 amino acids after the signal peptide cleavage site, and, in some cases, an EER motif (Stassen *et al*., 2013). The C‐terminal effector domains of many *Phytophthora* RXLR effectors contain conserved W, Y and L sequence motifs (Jiang *et al*., 2008). Crystal structure analysis of two seemingly sequence‐unrelated *Phytophthora* effectors revealed a conserved α‐helical fold, the WY domain, to which the previously identified W and Y sequence motifs map. The WY domain is present as a single module or repeat in approximately 44% of *Phytophthora* RXLR effectors (Boutemy *et al*., 2011).

Host‐translocated effectors may activate the immune system by interacting directly with NLRs or by the modification of host proteins that are ‘guarded’ by NLRs (Dangl and Jones, 2001; Van Der Biezen and Jones, 1998). NLRs can be classified into two main categories based on their N‐terminal domain: Toll/Interleukin‐1 receptor (TIR)‐type NLRs (TNLs) and coiled‐coil (CC)‐type NLRs (CNLs). The TIR and CC domains are followed by a nucleotide‐binding (NB) domain that forms the core of the resistance (R) protein, and C‐terminal leucine‐rich repeats (LRRs). Resistance is thought to be mediated predominantly by single NLRs. However, in recent years, the idea has emerged that NLRs can operate in pairs or networks (Wu *et al*., 2017), in which the ‘sensor’ NLR is responsible for effector detection and the ‘helper’ NLR initiates immune signalling (Césari *et al*., 2014b; Sukarta *et al*., 2016; Zhang *et al*., 2017). These pairs have evolved in both mono‐ and dicotyledonous plants. The genetically linked rice pair RGA4/RGA5 confers resistance against the fungal pathogen *Magnaporthe oryzae. *The ‘sensor’ CNL RGA5 negatively regulates the ‘helper’ CNL RGA4 in the absence of pathogen effectors to prevent RGA4‐mediated autoimmune responses (Cesari *et al*., 2014a). Negative regulation limits the expansion of NLR pairs as loss of the ‘sensor’ NLR would be detrimental for the plant. A network composed of multiple ‘sensor’ and ‘helper’ CNLs that operate through positive regulation is present in members of the asterids clade (e.g. coffee, pepper and tomato), but not in the rosids clade (e.g. Arabidopsis and soybean) (Wu *et al*., 2017). The asterid network is proposed to have evolved from a single NLR pair over 100 million years ago (Wu *et al*., 2017). Independently, the Arabidopsis ADR1 family evolved, consisting of three CNLs, that act redundantly as ‘helper’ NLRs to initiate immune signalling in cooperation with several CNL or TNL ‘sensors’ (Bonardi *et al*., 2011).

Genome analysis of lettuce (*Lactuca sativa*) cultivar Salinas identified 47 CNLs and 189 TNLs dispersed over all nine chromosomes of lettuce, although the majority of NLRs reside in five major resistance clusters (MRCs) on chromosomes 1, 2, 3, 4 and 8 (Christopoulou, McHale, *et al.*, 2015a). Lettuce MRCs contain downy mildew (*Dm*) resistance genes that confer monogenic protection against specific *Bremia lactucae* isolates (Christopoulou, McHale, *et al.*, 2015a; Giesbers *et al*., 2017; Parra *et al*., 2016). Substantial yield loss caused by the infection of lettuce with the oomycete *B. lactucae* is an important agricultural problem, and the introgression of *Dm* genes is a major focus of commercial breeding programmes. However, resistance mediated by newly introgressed *Dm* genes is rapidly broken by constantly evolving *B.* *lactucae* isolates. Wild lettuce species, such as *L. saligna*, *L. serriola* and *L. virosa*, are exploited as sources of new resistance genes (Parra *et al*., 2016).

Large‐scale *Agrobacterium tumefaciens‐*mediated transient expression of effectors has proven to be a useful tool in the identification and dissection of effector recognition specificities and disease resistance genes (Giesbers *et al*., 2017; Stassen *et al*., 2013; Vleeshouwers and Oliver, 2014; Vleeshouwers *et al*., 2008; Wroblewski *et al*., 2009). Previously, the recognition of four *B. lactucae* effectors that were transiently expressed via *A. tumefaciens* in a *Lactuca* germplasm set was reported (Giesbers *et al*., 2017; Stassen *et al*., 2013). Effectors BLG01 (GKLR motif), BLN08 (no RXLR motif) and BLR31 (RXLR motif) were specifically recognized in multiple *L. saligna* accessions. BLR31 recognition also co‐segregated with resistance to *B. lactucae* race 24 (Bl:24) and was mapped to MRC2 (Giesbers *et al*., 2017). Interestingly, the recognition of BLG01 and BLN08 was not linked to resistance to the effector‐producing race (Giesbers *et al*., 2017; Stassen *et al*., 2013). Finally, BLG03 (GKLR motif) is recognized in *L. sativa* cv. Amplus and UCDM2 that harbour the resistance gene *Dm2*. Although the recognition of BLG03 did not result in resistance against Bl:24, recognition did co‐segregate with resistance against *B. lactucae* race Bl:5 (Stassen *et al*., 2013).

In this study, 14 *B. lactucae* effectors were expressed in a lettuce germplasm set and the response of specific lettuce lines to three effectors is described. Interestingly, the response to RXLR effector BLR38 in *L. serriola *LS102 was mapped to two unlinked loci. Detailed genetic analysis revealed that the identified quantitative trait loci (QTLs) are both required for BLR38‐induced responses and confer gene dosage‐dependent HR induction. BLR38 recognition did not co‐segregate with resistance against race Bl:24, but was linked to resistance against multiple other *B. lactucae* races.

## Results

### BLN06 and BLR38 are recognized in *L. serriola* LS102

Previously, 16 novel *B. lactucae* effectors were identified from transcriptome sequences, although from this set only two effectors, BLN08 and BLR31, have been described so far (Giesbers *et al*., 2017). The remaining 14 effectors include the first identified *B. lactucae* Crinkler (BLC), eight effectors with the canonical RXLR motif (BLRs), one effector containing a QXLR motif (BLQ) and six effectors that only contain an EER‐like domain (BLN) (Tables 1 and S1, see Supporting Information). Putative WY domains were detected in two BLR effectors and two BLN effectors (Table 1).

**Table 1:** Overview of newly identified *Bremia lactucae* effectors.

Crinklers							
Effector	Protein length	Signal peptide	LXLFLAK motif		HVLVXXP motif		
			Motif	Start position	Motif	Start position	
BLC01	198	1–15	LRLFLAK	49	HVLVVP	114	

*RXLR‐like effectors*							
Effector	Protein length	Signal peptide	RXLR‐like motif		EER‐like motif		WY domains
			Motif	Start position	Motif	Start position	
BLN01	652	1–18	–	–	EER	48	Yes
BLN03	169	1–21	–	–	EER	52	No
BLN04	147	1–23	–	–	EER	59	No
BLN05	491	1–18	–	–	EER	50	No
BLN06	502	1–20	–	–	EER	51	Yes
BLQ04	531	1–15	QILR	27	EER	54	No
BLR32	146	1–22	RLLR	44	DER	56	No
BLR33	270	1–22	RRLR	50	DER	64	No
BLR35	514	1–16	RSLR	47	EER	58	No
BLR36	476	1–23	RALR	55	EER	68	Yes
BLR37	814	1–33	RRLR	42	–	–	Yes
BLR38	260	1–18	RLLR	46	–	–	No
BLR40	145	1–22	RRLR	44	EER	56	No

**Figure 1:**
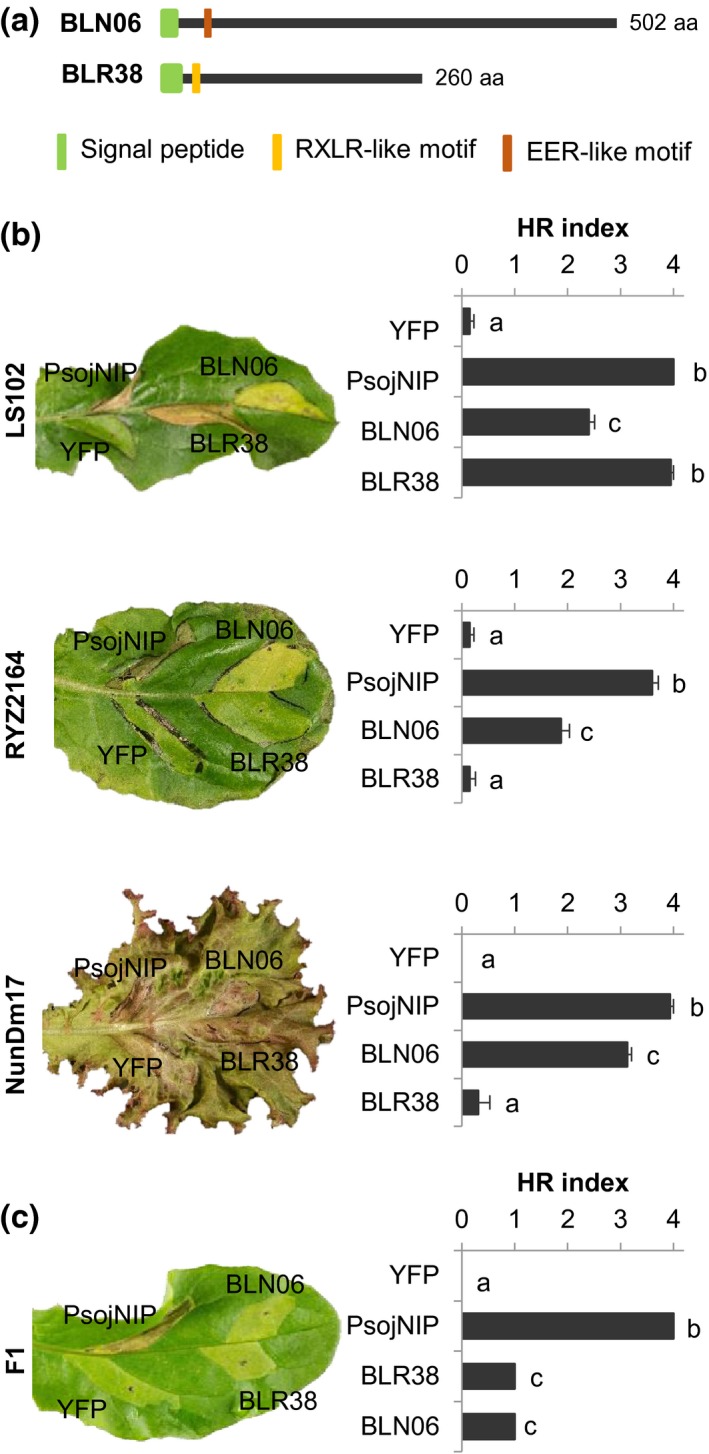
BLN06 and BLR38 are recognized in specific lettuce lines. (a) Schematic representation of *Bremia lactucae *effectors BLN06 and BLR38. aa, amino acid. (b) BLR38 and BLN06 are both recognized in *Lactuca serriola* LS102, and BLN06 is also recognized in *L. sativa* cv. RYZ2164 and NunDm17. Hypersensitive responses (HRs) were scored 5 days after infiltration of *Agrobacterium*. Bars represent the mean + standard error (SE) from 16–20 infiltration sites. Statistical differences were assessed using analysis of variance (ANOVA) with *post‐hoc *Tukey testing. YFP, yellow fluorescent protein. (c) Hypersensitive responses were scored 5 days after infiltration of *Agrobacterium* in F1 plants of *L. serriola* LS102 ×*L. sativa *GreenTowers. Bars represent the mean + SE from eight infiltration sites. [Colour figure can be viewed at wileyonlinelibrary.com]

To determine whether these 14 effectors are specifically recognized in lettuce, coding sequences were amplified starting from the position corresponding to the predicted signal peptide cleavage site, cloned and transiently expressed in a lettuce germplasm set consisting of 158 accessions and lines using *A. tumefaciens*. After the initial large‐scale screen (Table S2, see Supporting Information), effectors that triggered necrosis or chlorosis were validated in a second screen and responses to three effectors were confirmed (Table S3, see Supporting Information). Effector BLR40 triggered a robust HR in *L. sativa* cv. Design (Fig. S1, see Supporting Information) that is resistant to multiple *B. lactucae* races, including Bl:24 (Naktuinbouw, www.naktuinbouw.nl). Effectors BLN06 and BLR38 both induce a response in *L. serriola* LS102 (Fig. 1a,b), although there are several differences between the recognition of BLN06 and BLR38. First, the response to BLN06 was visible as chlorosis and was considerably weaker than the response to BLR38, which induced strong necrosis. Second, BLN06 was also recognized in *L. sativa* NunDm17 and RYZ2164, two lettuce cultivars that do not respond to BLR38 (Fig. 1b). Furthermore, *BLN06* and *BLR38* encode proteins of 502 and 260 amino acids, respectively, that share sequence homology with other *B. lactucae* effectors (Fig. S2, see Supporting Information), but not with each other. Finally, C‐terminal yellow fluorescent protein (YFP) fusion proteins of BLN06 and BLR38 showed a different subcellular localization pattern *in planta*. BLR38 contains a predicted nuclear localization signal (Kosugi *et al*., 2009) between positions 136 and 148, and, accordingly, BLR38‐YFP was exclusively present in the nucleus (Fig. S3a, see Supporting Information). In contrast, BLN06‐YFP and BLR40‐YFP accumulated in the plasma membrane (Fig. S3a). The recognition of BLN06‐YFP and BLR38‐YFP in *L. serriola* LS102 was not significantly different from untagged versions (Fig. S3b). Taken together, these data suggest that the recognition of BLN06 and BLR38 is mediated by different *R* genes.

### Expression of *BLN06, BLR38* and *BLR40* during infection

*BLN06*, *BLR38* and *BLR40* transcripts were originally identified in lettuce seedlings infected with *B. lactucae* race Bl:24, indicating that these effectors are expressed during infection. To determine whether the expression of *BLN06*, *BLR38* and *BLR40* varies at different infection stages, *L. sativa *cv. Olof seedlings were infected with race Bl:24 and samples were taken at 3 h and 1, 3 and 6 days after inoculation. *Bremia lactucae* biomass increased rapidly during this period and an approximately six‐fold higher transcript abundance of *B. lactucae ACTIN* compared with lettuce *ACTIN* was observed at 6 days after inoculation (Fig. S4, see Supporting Information). From 6 days after inoculation onwards, sporulation occurred on susceptible cotyledons. The relative abundance of *BLN06*, *BLR38* and *BLR40* transcript compared with *B. lactucae ACTIN* decreased slightly during the time course (Fig. S4), but did not show signs of strong down‐regulation. Thus, the transcription of these effectors could not be associated with specific infection stages.

### Recognition of BLR38 requires two unlinked loci

To determine whether the recognition of BLN06 and BLR38 is inherited as a dominant trait, *L. serriola* LS102 was crossed with *L. sativa* cv. GreenTowers, which is known to lack *R* genes to *B. lactucae* (Parra *et al*., 2016) and does not recognize BLN06 or BLR38. Transient *Agrobacterium*‐mediated expression of *BLN06* and *BLR38* in the resulting F1 plants and F2 population led to two striking observations. First, BLN06 responsiveness segregated in an unresponsive (28.7%), chlorotic (64.8%) and necrotic (6.5%) fraction in the F2 population, whereas only chlorosis was observed in F1 plants (Fig. 1c) and *L. serriola *LS102 (Fig. 1b). Second, expression of *BLR38* in F1 plants resulted consistently in intermediate response levels (i.e. chlorosis) (Fig. 1c), which is indicative of a semi‐dominant trait. In the F2 population, responsiveness segregated in an unresponsive (47.2%), chlorotic (21.3%) and necrotic (31.5%) fraction. Subsequent chi‐squared analysis did not support the hypothesis of a single semi‐dominant gene responsible for BLR38 recognition (tested ratio, 1 : 2 : 1; *χ* ^2^ = 37.8; df = 2; 95% confidence cut‐off *χ* ^2^ ≤ 5.991), suggesting that the recognition of this effector is genetically more complex.

**Figure 2:**
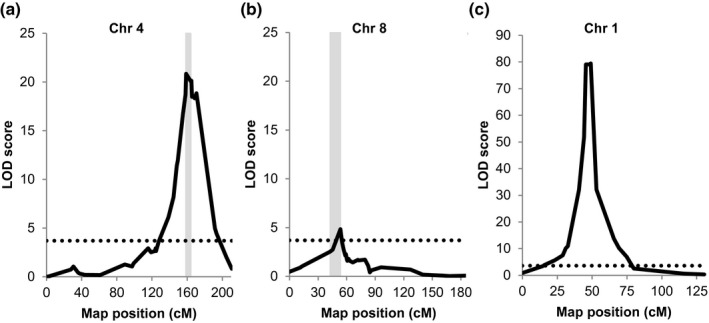
Recognition of BLR38 maps to loci on chromosomes (chr) 4 and 8, whereas resistance to *Bremia lactucae* Bl:24 maps to chr 1. Recognition of BLR38 maps to a locus at the bottom of chr 4 (a) and at the top of chr 8 (b). The smallest mapping intervals required for the recognition of BLR38 are depicted with vertical grey bars. (c) Resistance to Bl:24 in an F2 population of *Lactuca serriola* LS102 × *L. sativa* GreenTowers maps to the *Dm17* locus on chr 1. The significance threshold for the logarithm of the odds (LOD) scores is depicted as a broken horizontal line.

**Figure 3:**
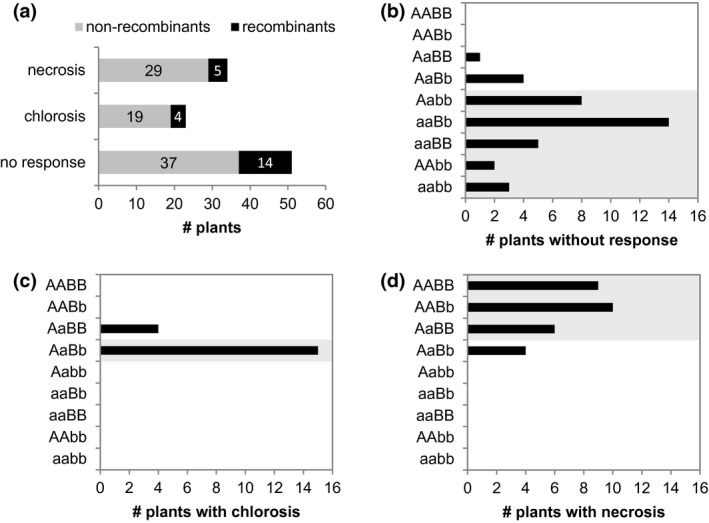
Gene dosage is an important factor in BLR38 recognition. BLR38 was transiently expressed using *Agrobacterium* in an F2 population of *Lactuca serriola* LS102 × *L. sativa* GreenTowers. (a) BLR38 recognition in the whole F2 population and the subpopulation without recombinants. Recombinants are defined here as plants with recombinations within the smallest mapping interval on chromosome (chr) 4 and/or chr 8 delineated by mLT19011 and mLT6534 on chr 4 and mLT9544 and mLT4241 on chr 8. (b–d) The distribution of non‐recombinants by genotype and response to transient expression of BLR38. Grey blocks indicate the genotypes most frequently observed to result in no response (b), chlorosis (c) and necrosis (d) on transient expression of BLR38. A and B, *L. serriola* LS102 genotype locus chr 4 and chr 8, respectively; a and b, *L. sativa* GreenTowers genotype locus chr 4 and chr 8, respectively.

To map loci mediating the recognition of BLN06 and BLR38, QTL analysis was performed on the F2 population. No significant loci were identified for BLN06 recognition, which could be caused by the weaker plant response that is more difficult to phenotype, and/or by complex and polygenic genetics underlying effector recognition and response. In contrast, mapping of BLR38 recognition revealed a QTL at the bottom of chromosome (chr) 4 with a logarithm of odds (LOD) score of 21 (Fig. 2a) and a weaker QTL at the top of chr 8 with a LOD score of 5 (Fig. 2b), which explain 56.3% and 17.5%, respectively, of the variance in this F2 population. The substantial difference in LOD score between the QTLs on chr 4 and chr 8 may reflect an unequal contribution of the QTLs to BLR38 recognition. To explore this, the smallest mapping intervals (Fig. 2a,b) were determined (for details, see Experimental procedures). Markers mLT19011 and mLT6534 on chr 4 and mLT9544 and mLT4241 on chr 8 delineated the smallest mapping intervals that span 18.7 and 8.1 Mbp, respectively, in *L. sativa* cv. Salinas reference genome v8. A simplified genotype based only on these markers could be assigned to 85 of the 108 F2 plants using ‘A’ and ‘B’ for the *L. serriola *LS102 alleles at chr 4 and chr 8, respectively, and ‘a’ and ‘b’ for the *L. sativa* GreenTowers alleles. As a result of recombinations within the selected mapping intervals, the other 23 plants were excluded from further analysis. Clear differences in genotype distribution per BLR38 response class were observed. In the absence of at least one of the *L. serriola* LS102 loci at either chr 4 and/or chr 8 (aabb, AAbb, aaBB, aaBb, Aabb), plants were, without exception, unresponsive to BLR38 (Fig. 3b). *Agrobacterium* infiltration sites of heterozygous (AaBb) plants turned predominantly chlorotic (15/23), but also no response (4/23) and necrosis (4/23) were observed (Fig. 3c). As expected, a necrotic response was observed in plants homozygous for the *L. serriola* LS102 alleles at both loci (Fig. 3d). Surprisingly, plants heterozygous for the *L. serriola *LS102 allele at chr 8 showed a strong response to BLR38 (AABb, 10/10 necrosis), whereas plants heterozygous at chr 4 showed a lower number of necrotic infiltration sites (AaBB, 6/11 necrosis), suggesting that the gene product of chr 4 is more rate limiting than that of chr 8. The observed requirement for both loci was confirmed by the segregation ratio in the F2 population, where the response segregates in a 7 : 4 : 5 (unresponsive : chlorosis : necrosis) ratio (tested ratio, 7 : 4 : 5; *χ* ^2^ = 0.89; df = 2; 95% confidence cut‐off *χ* ^2^ ≤ 5.991). In conclusion, the *L. serriola* LS102 loci on chr 4 and chr 8 are semi‐dominant and are both required to mediate the recognition of BLR38.

### F3 analysis confirms a digenic model for BLR38 recognition

To further substantiate the digenic model, responsiveness to BLR38 was assessed in F3 progeny of selected F2 plants. As expected, F3 families #6 and #28, which lack the *L. serriola* LS102 allele at chr 4 or chr 8 (F2 genotypes aaBB and AAbb), remained unresponsive to BLR38, whereas a severe necrotic response was observed in all F3 plants from family #27, which is homozygous for the *L. serriola* LS102 allele at both chromosomes (F2 genotype AABB) (Table 2). F3 progeny of heterozygous (AaBb) F2 parents are expected to segregate similarly to the F2 population in a 7 : 4 : 5 (unresponsive : chlorosis : necrosis) ratio. Four families indeed segregated in three response classes, but only F3 families #108 and #113 segregated in the expected 7 : 4 : 5 ratio. In F3 families #69 and #117, a higher fraction of unresponsive (observed, 54% and 52%; expected, 43.75%) infiltration sites was observed and a lower fraction of chlorotic sites (observed, 8% and 14%; expected, 25%) (Table 2). This tilting towards the extremes may be related to the fixation of modifier loci as a result of a reduction in heterozygosity from 50% in F2 plants to 25% in F3 plants. Altogether, the F3 analysis supports a digenic model to describe BLR38 recognition.

**Table 2:** Response of selected *Lactuca serriola* LS102 × *L. sativa* GreenTowers F3 families to the transient expression of BLR38.

F3 family	F2 genotype*	Expected (unresponsive : chlorotic : necrotic)	Observed (unresponsive : chlorotic : necrotic)	*χ* ^2^ (cut‐off = 5.991)
*Non‐segregating families for BLR38 recognition*				
#6	AAbb	16 : 0 : 0	28 : 1 : 0	0.75
#28	aaBB	16 : 0 : 0	30 : 0 : 0	0
#27	AABB	0 : 0 : 16	0 : 0 : 29	0
*Segregating families for BLR38 recognition*				
#69	AaBb	7 : 4 : 5	14 : 2 : 10	15.98
#108	AaBb	7 : 4 : 5	13 : 5 : 6	4.42
#113	AaBb	7 : 4 : 5	14 : 5 : 10	3.21
#117	AaBb	7 : 4 : 5	15 : 4 : 10	6.81

* A and B, *L. serriola* LS102 genotype locus chromosome 4 and chromosome 8, respectively; a and b, *L. sativa* GreenTowers genotype locus chromosome 4 and chromosome 8, respectively.

### Resistance to Bl:24 in *L. serriola *LS102 is independent of BLN06 or BLR38 recognition and mediated by *Dm17*

**Figure 4:**
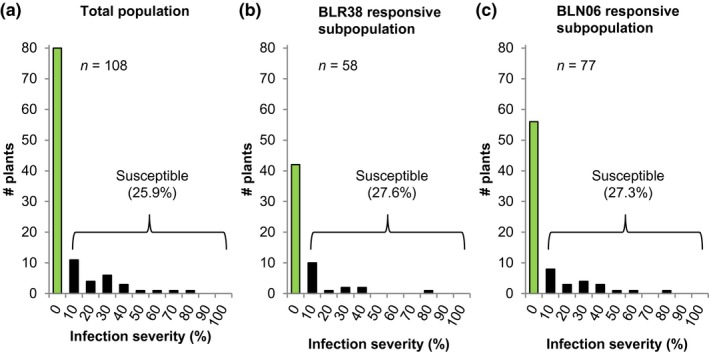
BLN06 and BLR38 recognition is independent of resistance to Bl:24. (a) Resistance to Bl:24 in an F2 population of *Lactuca serriola* LS102 × *L. sativa* GreenTowers. Leaf discs were infected with Bl:24 (60 spores/µL) and the percentage of leaf disc surface covered in sporangiophores was scored 8 days after inoculation. Infection classes start at 0% and increase in 10% increments. (b,c) Resistance to Bl:24 in F2 plants that scored ≥1 (on a scale from 0 = no response to 4 = severe necrosis) on transient expression of BLR38 (b) or BLN06 (c) using *Agrobacterium*. Two infiltration sites per plant were scored 4–6 days after infiltration with the highest score considered leading. Green bars represent fully resistant plants (no sporulation). [Colour figure can be viewed at wileyonlinelibrary.com]

The BLR38‐ and BLN06‐recognizing line *L. serriola* LS102 is resistant to infection by *B. lactucae* race Bl:24 from which *BLR38* and *BLN06* were cloned. To test whether recognition of BLN06 or BLR38 is genetically linked to resistance, disease assays with *B. lactucae *race Bl:24 were performed on the *L. serriola *LS102 × *L. sativa* GreenTowers F2 population. Eighty of 108 F2 plants were resistant to Bl:24, indicating the presence of a single dominant resistance gene (tested ratio, 3 : 1; *χ* ^2^ = 0.05; df = 1; 95% confidence cut‐off *χ* ^2^ ≤ 3.84). Resistance to Bl:24 segregated in the BLN06 and BLR38 responsive subpopulations in the same 3 : 1 ratio as observed for the F2 population as a whole (Fig. 4). This demonstrates that BLN06 and BLR38 recognition is not linked to resistance to Bl:24.

The BLN06‐recognizing lines *L. serriola* LS102 and *L. sativa* NunDm17 possess *Dm17,* which confers resistance against races Bl:1–Bl:7, Bl:10–Bl:26 and Bl:28 (Parra *et al*., 2016). To confirm that the observed single dominant resistance against Bl:24 in the *L. serriola *LS102 × *L. sativa* GreenTowers F2 population was mediated by *Dm17*, QTL analysis was performed. Disease resistance to Bl:24 was mapped to a single, highly significant peak at the top of chromosome 1 with a LOD score of 79 (Fig. 2c), corresponding to the reported location of *Dm17* (Maisonneuve *et al*., 1994). Thus, Bl:24 is expected to contain an additional, unidentified effector that triggers *Dm17*‐mediated resistance in our F2 population.

### BLR38 recognition is linked to resistance to other *B. lactucae *races

**Figure 5:**
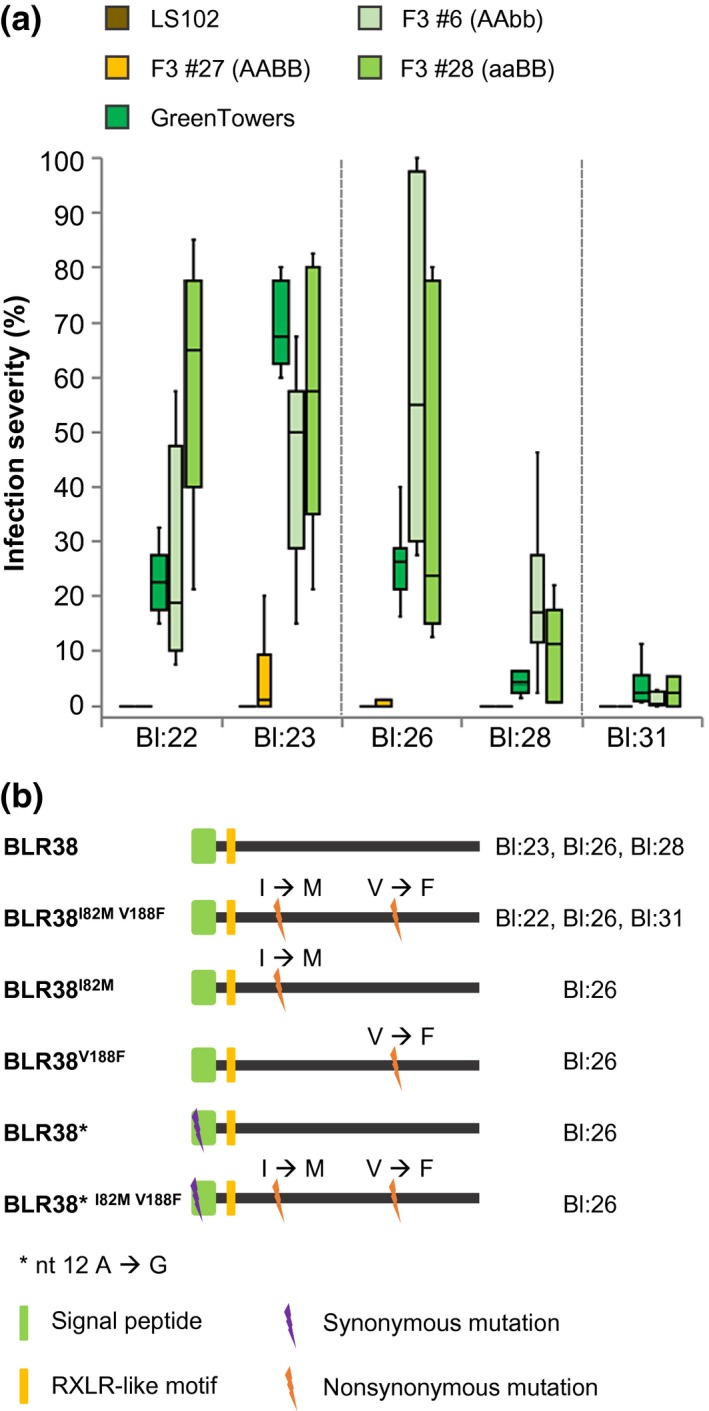
Recognition of BLR38 induces resistance against multiple *Bremia lactucae* races. (a) Susceptibility of BLR38‐recognizing *Lactuca serriola* LS102 × *L. sativa* GreenTowers F3 family #27 and BLR38‐non‐recognizing F3 families #6 and #28 to *B. lactucae* races Bl:22, Bl:23, Bl:26, Bl:28 and Bl:31. Data from three independent experiments with distinct batches of plants and *B. lactucae* races are depicted separated by grey lines. Leaf discs were punched from seven plants per line or family and were sprayed with 80–100 spores/µL of Bl:22 or Bl23, ~150 spores/µL of Bl:26 or Bl:28, or 100 spores/µL of Bl:31, and scored at 10–11 days after inoculation. (b) Allelic variation of *BLR38* in *B. lactucae* races. RNA was isolated from infected seedlings and *BLR38* was cloned using 5′ and 3′ untranslated region (UTR) binding primers. The top allele represents the reference sequence originating from Bl:24. nt, nucleotide. [Colour figure can be viewed at wileyonlinelibrary.com]

Previously, the recognition of effector BLG03 was found to co‐segregate with resistance against *B. lactucae *race Bl:5, but not Bl:24 (Stassen *et al*., 2013). Thus, the absence of linkage between BLR38 recognition and resistance against Bl:24 did not preclude linkage between BLR38 recognition and resistance to other *B. lactucae* races. To explore this possibility, leaf disc assays were performed on F3 families with races Bl:22, Bl:23, Bl:26, Bl:28 and Bl:31. To exclude confounding effects of *Dm17 *in the disease assays, F3 families lacking *Dm17* were selected from crosses between *L. serriola* LS102 and *L. sativa* cv. GreenTowers (families #27, #6 and #28) and CobhamGreen. BLR38‐recognizing family #27 (F2 genotype AABB) displayed moderate to strong resistance against races Bl:22, Bl:23 and Bl:26 (average infection severity, 0.0%–4.7%), whereas the BLR38‐non‐recognizing F3 families #6 (F2 genotype aaBB) and #28 (F2 genotype AAbb) were susceptible to these three races (average infection severity, 26.6%–57.1%) (Fig. 5a). A similar trend was observed with races Bl:28 and Bl:31, even though the difference between the BLR38‐recognizing (average infection severity, 0.0%) and BLR38‐non‐recognizing (average infection severity, 1.2%–20.1%) families was much smaller because of the lower virulence of these *B. lactucae* races. The BLR38‐recognizing *L. serriola *LS102 × *L. sativa* cv. CobhamGreen F3 families were also moderately to highly resistant to all five tested races. BLR38‐non‐recognizing *L. serriola *LS102 × *L. sativa* cv. CobhamGreen F3 families were more susceptible, although infection levels varied (Fig. S5, see Supporting Information). In summary, these results demonstrate that the recognition of BLR38 is linked to resistance against races Bl:22, Bl:23, Bl:26, Bl:28 and Bl:31.

Allelic variation in effector genes between races of a pathogen species can affect recognition in the pathogen host. To determine whether allelic variation in *BLR38* is present in *B. lactucae *races, the coding sequence was amplified from races Bl:22, Bl:23, Bl:24, Bl:26, Bl:28 and Bl:31 using primers designed on the 5′ and 3′ untranslated regions (UTRs) of the Bl:24‐derived *BLR38* transcript. Races Bl:23, Bl:24 and Bl:28 were all homozygous for the Bl:24 allele. Races Bl:22 and Bl:31 were homozygous for a second allele with two single nucleotide polymorphisms (SNPs) in the effector domain, resulting in amino acid substitutions at position 82, isoleucine (I) to methionine (M), and position 188, valine (V) to phenylalanine (F). Surprisingly, six variants including the Bl:24 sequence and Bl:22/Bl:31 variant (encoding *BLR38* ^I82M V188F^) were identified in cDNA of Bl:26 (Fig. 5b). Yet, the effector variants with non‐synonymous SNPs were all still recognized in *L. serriola* LS102 (Fig. S6, see Supporting Information), suggesting that the identified alleles have not evolved to escape detection by the *L. serriola* LS102 resistance proteins.

### The effect of gene dosage on BLR38‐induced resistance

To further substantiate the linkage between BLR38 recognition and resistance against *B. lactucae*, a leaf disc assay with Bl:22 was performed on *L. serriola *LS102 × *L. sativa* GreenTowers F3 family #108, which segregates for the two loci required for BLR38 recognition. Individual F3 plants were tested for BLR38 recognition and genotyped using additional markers in the previously identified QTL intervals on chr 4 and chr 8. Mapping of either disease resistance or BLR38 recognition resulted in maximum LOD scores at the same marker positions on chr 4 and chr 8, confirming previous results. Maximum LOD scores associated with resistance were 3.3 on chr 4 and 7.6 on chr 8, whereas, for BLR38 recognition, they were 6.4 on chr 4 and 12.2 on chr 8 (Fig. S7, see Supporting Information).

**Figure 6:**
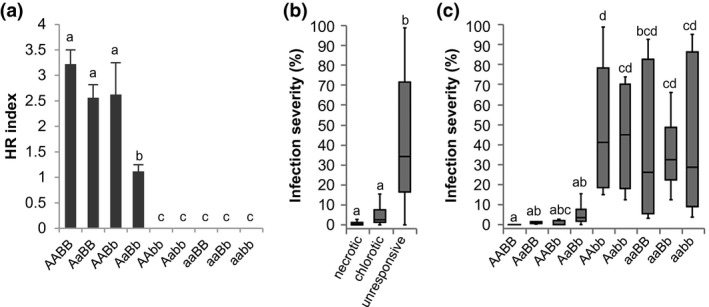
Gene dosage plays a minor role in resistance to race Bl:22 in BLR38‐recognizing plants. (a) Recognition of BLR38 in *Lactuca serriola* LS102 × *L. sativa* GreenTowers F3 family #108. Genotypes were assigned based on markers mLT35950571 and mLT105577576 on chromosome (chr) 4 and mLT48854237 and mLT4241 on chr 8. Hypersensitive responses (HRs) were scored 5–6 days after *Agrobacterium*‐mediated transient transformation. On the same plants, a leaf disc assay was performed with Bl:22 (100 spores/µL) and the percentage of leaf disc surface covered in sporangiophores was scored 10 days after inoculation. (b) Plants were grouped on the basis of the highest score of two infiltration sites and were divided over three response classes: unresponsive (score, 0), chlorotic (score, 0.5–2) or necrotic (score, ≥2.5). (c) Plants were grouped on the basis of genotype. Statistical differences were assessed using analysis of variance (ANOVA) with *post‐hoc *Tukey testing.

To determine whether resistance against Bl:22 is dependent on gene dosage, simplified genotypes were assigned to 88 of 115 plants. These plants were not recombinant between markers mLT35950571 and mLT105577576 on chr 4 and markers mLT48854237 and mLT4241 on chr 8 that flank the reduced smallest mapping intervals (Fig. S7). In all five susceptible (average infection severity, 39.8%–48.0%) F3 genotypes, the *L. serriola* LS102 allele was absent on chr 4 and/or chr 8 (Fig. 6c). These plants were also unresponsive to BLR38 (Fig. 6a). Heterozygous plants (AaBb) were moderately resistant (average infection severity, 6.5%). AABB, AaBB and AABb plants were highly resistant (average infection severity, 0.03%–1.34%) (Fig. 6c). When plants were grouped by BLR38 response class, BLR38‐unresponsive plants were significantly more susceptible to Bl:22 (average infection severity, 42.1%) than necrotic (average infection severity, 0.9%) and chlorotic (average infection severity, 5.7%) responders (Fig. 6b). However, the susceptibility of the chlorotic responders was not significantly different from that of the necrotic responders. Thus, gene dosage plays a more limited role in resistance to *B. lactucae* than in BLR38 effector recognition.

### The QTLs are located near MRCs

**Figure 7:**
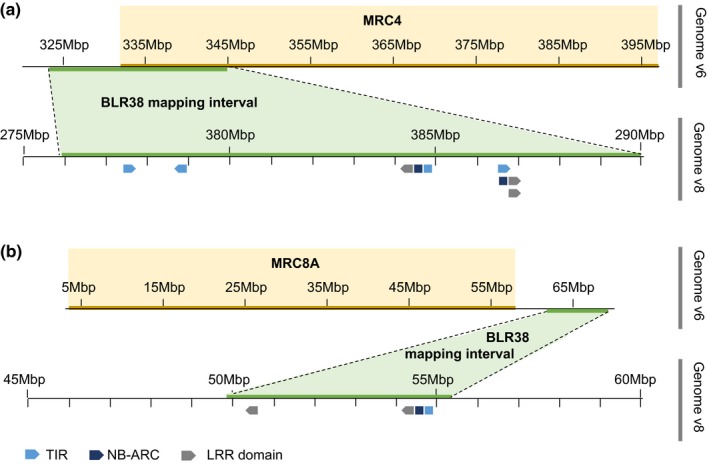
Graphical representation of the mapping intervals conferring BLR38 recognition relative to major resistance clusters (MRCs). The top panels show the position of MRC4 (a) and MRC8A (b) as reported by Christopoulou *et al*. (2015a, 2015b). The positions of the smallest mapping intervals for BLR38 recognition are depicted according to *Lactuca sativa* cv. Salinas genomes v6 and v8. The bottom panels indicate the positions of genes containing Toll/Interleukin‐1 receptor (TIR) (light blue), NB‐ARC (nucleotide‐binding adapter shared by APAF‐1, R proteins and CED‐4; dark blue) and leucine‐rich repeat (LRR) (grey) domains (not to scale). [Colour figure can be viewed at wileyonlinelibrary.com]

The majority of resistance loci in cultivated lettuce are located on five MRCs on chr 1, 2, 3, 4 and 8 (Christopoulou, Wo, *et al.*, 2015b; Christopoulou, McHale, *et al.*, 2015a; McHale *et al*., 2009). The position of the loci mediating BLR38 recognition was compared with the position of MRCs in the *L. sativa* cv. Salinas reference genome v6 (Christopoulou, Wo, *et al.*, 2015b; Christopoulou, McHale, *et al.*, 2015a) to determine whether there is overlap with resistance gene clusters. The smallest mapping interval on chr 4 partially overlapped with the upstream border of MRC4 (Fig. 7a), whereas the smallest mapping interval on chr 8 was located just downstream of MRC8A (Fig. 7b). Based on genotyping of F3 family #108, the smallest mapping intervals on chr 4 and chr 8 spanned 14.2 and 5.5 Mbp (genome v8), respectively (Reyes‐Chin‐Wo *et al*., 2017). Genes within these intervals were screened for the presence of TIR, NB‐ARC (nucleotide‐binding adapter shared by APAF‐1, R proteins and CED‐4) and LRR domains which are characteristic of NLRs. Both regions contain NLRs with TIR domains (Table S4, see Supporting Information). Thus, it is probable that the corresponding regions in the *L. serriola *LS102 genome also contain NLRs that could cooperatively mediate BLR38 recognition.

## Discussion

In this study, the recognition of *B. lactucae* effectors BLN06 and BLR38 in the wild lettuce accession *L. serriola* LS102 is described. Although effector recognition is frequently associated with single dominant *R* gene loci (Flor, 1971; Michelmore and Wong, 2008; Vleeshouwers *et al*., 2008), the recognition of BLR38 required two unlinked loci that displayed incomplete dominance, resulting in a 7 : 4 : 5 (unresponsive : chlorotic : necrotic) ratio in the F2 population. Similarly, a study on non‐host resistance in pepper against *Phytophthora infestans* found that, although the loci involved in the recognition of five *P. infestans *effectors were dominant, the response to PexRD24, PexRD46 and PexRD50 segregated in a 7 : 9 (unresponsive : necrotic) ratio, consistent with two unlinked loci whose gene products interacted complementarily (Lee *et al*., 2014). Yet, our data contrasted with previously characterized *B. lactucae* effector responses that mapped to single loci (Giesbers *et al*., 2017; Stassen *et al*., 2013). In addition, lettuce responses to multiple bacterial type III effectors from *Pseudomonas* and *Ralstonia* pathovars segregated as single dominant loci (Wroblewski *et al*., 2009).

The recognition of BLR38 was not genetically linked to resistance against *B. lactucae* race Bl:24, from which the effector was cloned, but was linked to resistance against races Bl:22, Bl:23, Bl:26, Bl:28 and Bl:31. Six *BLR38* alleles were identified in these *B. lactucae* races, including the Bl:24‐derived allele. The three BLR38 variants with amino acid substitutions were still recognized in *L. serriola* LS102, indicating that *BLR38* allelic variation in these races did not evolve to overcome resistance mediated by *L. serriola* LS102 encoded genes. In addition, the recognition of effectors BLG01, BLG03, BLN06 and BLN08 was not genetically linked to resistance against Bl:24, whereas the recognition of BLR31 did provide resistance to Bl:24 (Giesbers *et al*., 2017; Stassen *et al*., 2013). It has been postulated previously that *B. lactucae* expresses additional effectors that suppress effector recognition or downstream immune signalling (Giesbers *et al*., 2017; Stassen *et al*., 2013). Race Bl:24 is thought to suppress the recognition or response to at least five Bl:24‐derived effectors, but not to that recognized by *Dm17*. This suggests that specific pathways involved in effector‐triggered immunity (ETI) are suppressed by Bl:24, possibly by newly evolved and yet unidentified effectors. Similarly, *P. infestans* RXLR effector PexRD2 and *Xanthomonas euvesicatoria* type III effector XopQ target distinct branches of the mitogen‐activated protein kinase (MAPK) signalling pathway involved in the activation of ETI, and are thereby able to suppress HR induced by various effectors (King *et al*., 2014; Teper *et al*., 2013).

Despite a clear effect of gene dosage on BLR38 recognition in F2 and F3 plants, the observed difference in infection severity between plants homozygous or heterozygous for the *L. serriola* LS102 loci was not significant. Gene dosage‐dependent resistance against *B. lactucae* has been reported previously for *Dm6 *(Crute and Norwood, 1986) and *Dm17* under extreme disease pressure (Maisonneuve *et al*., 1994). Indeed, gene dosage effects may be more pronounced under specific circumstances (e.g. at the seedling stage) or at high disease pressures, when transcription from a single allele is more likely to become the rate‐limiting determinant of the immune response than under low disease pressure circumstances. Incomplete dominance of resistance to *BLR38*‐expressing *B. lactucae* races could be apparent under specific environmental conditions and/or disease pressures. The gene dosage effects observed in BLR38 transient expression assays strongly suggest that transcript levels of the loci conferring HR to this effector are indeed rate limiting under these circumstances, resulting in chlorosis in heterozygous plants.

The loci mediating BLR38 recognition were mapped in a segregating F2 and F3 population to chr 4 and chr 8. The equivalent intervals in the *L. sativa *cv. Salinas reference genome contain several genes that encode proteins associated with resistance as a result of the presence of TIR, NB‐ARC and LRR domains. Thus, *NLRs* are candidate genes for the mediation of BLR38 recognition and induction of disease resistance against several *B. lactucae* races. Multiple NLRs have been reported to operate in pairs (Ashikawa *et al*., 2008; Saucet *et al*., 2015; Wu *et al*., 2016; Yuan *et al*., 2011), and the two components are postulated to have distinct functions: one acts as a ‘sensor’ and is responsible for the recognition of the pathogen effector or effector‐modified plant protein, whereas the other NLR acts as a ‘helper’ or ‘executor’ and mediates the activation of downstream signalling pathways (Sukarta *et al*., 2016; Wu *et al*., 2017). The ‘sensor’ NLR repertoire of a plant is highly divergent to allow for the identification of a broad range of pathogens. On the contrary, ‘helper’ NLRs benefit from a certain amount of redundancy and conservation to ensure robustness in the immune system (Wu *et al*., 2017). To determine whether the loci mediating BLR38 recognition constitute a ‘sensor’ and ‘helper’ NLR pair, sequencing of the corresponding regions in the *L. serriola* LS102 genome is required. Then, candidate genes could be amplified, cloned and transiently co‐expressed with BLR38 to complement F3 families that lack the *L. serriola* LS102 allele at chr 4 or chr 8 (aaBB or AAbb). Alternatively, the loci mediating BLR38 recognition could constitute an R protein and an effector target. In this model, the R protein mediates indirect recognition of the effector by monitoring an effector target with a function in host susceptibility (‘guardee’) or an effector target that has no function in the host other than activating the guarding R protein through interaction with the effector (‘decoy’) (Van der Hoorn and Kamoun, 2008).

Our large‐scale transient expression assay in lettuce germplasm showed that BLN06 is not only recognized in *L. serriola* LS102, but also in *L. sativa* NunDm17 and RYZ2164. Although the link with resistance gene *Dm17*, which is present in all three lines, seemed obvious, BLN06 responsiveness and *Dm17 *were not genetically linked. BLN06 responsiveness segregated in a 3 : 1 (responsive : unresponsive) ratio in the *L. serriola* LS102 × *L. sativa* GreenTowers F2 population, suggesting a simple single gene model; yet, interval mapping failed to identify significant QTLs. Unexpectedly, a small number (6.5%) of *Agrobacterium* infiltration sites in F2 plants displayed necrosis, a much stronger response than observed in *L. serriola* LS102 or in F1 plants. Transgressive segregation, i.e. the offspring of a cross displays a more extreme phenotype than the parents, is generally attributed to complementary gene action amongst multiple loci. The extreme phenotypes can arise when alleles of opposing effects, present in the parents, are recombined in the offspring and result in allele combinations with an additive effect (Bell and Travis, 2005; Nelson *et al*., 2018; Rieseberg *et al*., 1999). As allele combinations can be selected for, these can become fixed in populations, thereby making transgressive phenotypes heritable (Rieseberg *et al*., 1999). It is possible that this occurred in *L. sativa* NunDm17, which could explain our finding that *Agrobacterium* infiltration sites in *L. sativa* NunDm17 (derived from *L. serriola* LS102; Maisonneuve *et al*., 1994; Parra *et al*., 2016) were predominantly necrotic. Thus, the recognition of BLN06 may be a more complex trait than anticipated, and further research is required to determine the number of loci involved and their individual contributions.

## Experimental Procedures

### Plant growth conditions and *B. lactucae* maintenance

Lettuce seed germination as well as *B. lactucae* maintenance and disease assays were performed under short‐day growth conditions [9 h of light (100 µE/m^2^/s)] at 16 °C. To maintain high humidity during infection, a tray with infected plant material was closed with a transparent lid, a dish with water was placed inside and the edges were sealed with tape. *Nicotiana benthamiana* plants and germinated lettuce seedlings were grown under long‐day conditions (16 h of light, 70% humidity) at 21 °C. *Lactuca serriola *LS102 is also known as LS‐102 and corresponds to accession number CGN24780 at the Centre for Genetic Resources (Wageningen, the Netherlands).

### Candidate effector identification and cloning

To expand the *B. lactucae* effectorome, Illumina‐based RNA sequencing (RNAseq) was performed on mRNA derived from *B. lactucae* spores and *B. lactucae*‐infected lettuce seedlings, as described previously (Giesbers *et al*., 2017). To identify novel effector candidates, proteins in the secretome (Giesbers *et al*., 2017) were analysed with a Perl script using regular expressions for RXLR‐like, dEER and LXLFLAK motifs. Second, a homology search of the secretome against a database composed of known effector sequences from *B. lactucae*, *Phytophthora cinnamomi*, *P. infestans*, *P. parasitica*, *P. ramorum*, *P. sojae*, *P. andina*, *Pseudoperonospora cubensis *and *Hyaloperonospora arabidopsidis* was conducted. WY motifs were identified using a hidden Markov model (HMM) (Boutemy *et al*., 2011) and, after manual inspection, an *E*‐value cut‐off of 0.001 for the best motif within an effector was set. Effector coding sequences were polymerase chain reaction (PCR) amplified from *B. lactucae *race 24 cDNA from the signal peptide cleavage site and cloned into pENTR/D‐TOPO (Invitrogen, Carlsbad, CA, USA) with a new start codon. The resulting entry clones were recombined with the binary vector pK2GW7 using LR clonase to generate untagged effector constructs suitable for transformation into *A. tumefaciens* strain C58C1 (pGV2260), according to Stassen *et al*. (2013). The primers used for cloning are listed in Table S5 (see Supporting Information).

### Accession numbers

Effector coding sequences were deposited under the following GenBank numbers: BLR32 (MG686566), BLR33 (MG686567), BLR35 (MG686568), BLR36 (MG686569), BLR37 (MG686570), BLR38 (MG686571), BLR40 (MG686572), BLC01 (MG686573), BLN01 (MG686574), BLN03 (MG686575), BLN04 (MG686576), BLN05 (MG686577), BLN06 (MG686578) and BLQ04 (MG686579).

### Effector recognition assays in lettuce

*Agrobacterium tumefaciens* strains harbouring *B. lactucae* effector constructs were grown in Luria–Bertani medium o/n at 28 °C and 200 rpm. Cells were spun down for 10 min at ~3000 g rpm and resuspended in induction medium [8.5 g/L Na_2_HPO_4_.2H_2_O, 3 g/L KH_2_PO_4_, 0.5 g/L NaCl, 1 g/L NH_4_Cl, 1% (w/v) glucose, 50 µm acetosyringone, 50 µg/mL rifampicin, 50 µg/mL carbenicillin and 100 µg/mL spectinomycin] to an optical density at 600 nm (OD_600_) < 1. Cultures were incubated for 3–4 h at 28 °C and 200 rpm. Cells were spun down for 10 min at ~3000 g rpm and resuspended in infiltration medium [0.5 × MS salts, 10 mm MES, 0.5% (w/v) sucrose, 0.5% (w/v) fructose, 150 µm acetosyringone, pH 5.6] to an OD_600_ of 0.4. Leaves of 3‐week‐old lettuce plants were infiltrated with bacterial suspensions and scored 3–5 days after infiltration. Infiltration sites were scored from 0 (no chlorosis) to 4 (severe necrosis) (Fig. S8, see Supporting Information). Plants that clearly responded to the negative control (YFP) or failed to respond to the positive control [*Phytophthora sojae* necrosis‐inducing protein (PsojNIP)] (Qutob *et al.*, 2002) were left out of the analysis.

### RNA isolation

Seedlings were flash‐frozen in liquid nitrogen and ground using a mortar and pestle or the TissueLyser (Qiagen, Hilden, Germany) to a fine powder. Total RNA was extracted using the Spectrum Plant Total RNA kit (Sigma‐Aldrich, St. Louis, MO, USA) and treated with DNase I (ThermoFisher Scientific, Carlsbad, CA, USA) to remove genomic DNA. cDNA was synthesized using RevertAid H Minus Reverse Transcriptase (ThermoFisher Scientific, Carlsbad, CA, USA).

### Time course quantitative reverse transcription‐polymerase chain reaction (RT‐PCR)

Three to four‐day‐old *L. sativa* cv. Olof seedlings were sprayed with tap water or a spore suspension of *B. lactucae* race Bl:24 (≈100 spores/µL). Cotyledons were collected at 3 h and 1, 3 and 6 days after spraying. Relative transcript levels were determined in three biological replicates per experiment (each replicate being measured in two technical replicates) using SYBR Green qPCR Master mix (ThermoFisher Scientific, Carlsbad, CA, USA) and the ViiA7 Real‐Time PCR system (Applied Biosystems, Foster City, CA, USA). *Bremia lactucae* *ACTIN* transcript levels were normalized to *L. sativa* *ACTIN* transcript. Effector gene expression levels were normalized to *B. lactucae* *ACTIN*. The primers used for quantitative RT‐PCR are listed in Table S5.

### Leaf disc assays

Leaf discs were punched from 4‐week‐old lettuce plants, placed upside down in a transparent tray on four layers of soaked filter paper and sprayed with a spore suspension of *B. lactucae*. The spore density differed between experiments and races, and was 50–150 spores/µL. At 8–11 days after inoculation, the percentage of leaf disc area covered in sporangiophores was scored, unless indicated otherwise.

### Genotyping

Ten leaf discs (Ø = 6 mm) were punched per plant and placed in a 96‐well tray. Leaf discs were dried by placing a silica bag on top of the plate and applying a vacuum to the sealed package. DNA was isolated using the sbeadex maxi plant kit (LGC Genomics, Berlin, Germany) and verified on a NanoDrop‐8000 spectrophotometer (ThermoFisher Scientific, Wilmington, DE, USA). Genotyping was carried out using KASP genotyping chemistry (LGC Genomics, Berlin, Germany) on Fluidigm chips. Data were analysed using Fluidigm SNP genotyping software. A genetic linkage map was constructed using JoinMap 4.0 software (Van Ooijen, 2006), and QTLs were detected using MapQTL 5.0 software (Van Ooijen, 2004).

To determine the smallest mapping interval on chr 4, BLR38‐unresponsive plants homozygous for *L. serriola* LS102 alleles at the QTL on chr 8 were selected. In these plants, BLR38 unresponsiveness was caused by homozygous *L. sativa* GreenTowers alleles on chr 4 at marker positions closely linked to BLR38 recognition. After delineating the interval on chr 4, the smallest mapping interval on chr 8 was determined with BLR38‐unresponsive plants that were homozygous for *L. serriola* LS102 on chr 4. For the markers defining the smallest mapping intervals on chr 4 and chr 8, the SNP with its flanking sequence is listed in Table S5.

### Sequencing of effector alleles

Primers were designed on the 5′ and 3′ UTRs of *BLR38* mRNA originating from Bl:24 (Table S5). *BLR38* was amplified using Phusion High‐Fidelity DNA polymerase from cDNA prepared from seedlings heavily infected with *B. lactucae* races Bl:22, Bl:23, Bl:24, Bl:26, Bl:28 or Bl:31. PCR products containing attB sites were recombined in a modified pGemTEasy vector containing the pDONR201 Gateway recombination site (pGemTEasy^mod^) in a BP clonase reaction. Per race, 10 transformed *Escherichia coli* DH5α colonies were selected for plasmid DNA isolation and subsequent Sanger sequencing. SNPs, identified by comparison with the Bl:24 *BLR38* reference sequence, were considered when detected in at least three independent sequences.

### Effector localization in *N. benthamiana*

Open‐ended constructs of BLN06, BLR38 and BLR40 were cloned into pGemTEasy^mod^. Entry clones were recombined with pB7YWG2 using LR clonase to generate constructs with the effector fused at the C‐terminus to YFP. An *A. tumefaciens *strain carrying pB7WGC2 [free cyan fluorescent protein (CFP)] was co‐infiltrated with strains harbouring effector fusion constructs resuspended in infiltration buffer (10 mm MES, 10 mm MgCl_2_ and 150 µm acetosyringone, pH 5.6) to an OD_600_ of 0.4 per *A. tumefaciens* strain in the leaves of 4–5‐week‐old *N. benthamiana* plants. Leaf sections were examined at 2–3 days after infiltration using a Zeiss LSM 700 (Jena, Germany) laser scanning microscope. Plant cell walls were stained by incubation for 7–10 min in 5 mg/mL propidium iodide (PI) solution. To distinguish membrane‐localized proteins from cytoplasmic proteins, cells were viewed in multiple focal planes to confirm the absence of cytoplasmic strands. CFP, YFP and PI were excited at 405, 488 and 555 nm, respectively. Emitted light of both CFP and YFP was captured using a 490–555‐nm band‐pass filter, and emitted light of PI was captured using a 560‐nm long‐pass filter.

## Supporting information

Fig. S1  BLR40 is specifically recognized in *Lactuca sativa* cv. Design. (a) Schematic representation of *B. lactucae *effector BLR40. (b) BLR40 is recognized in *L. sativa* cv. Design. Hypersensitive responses were scored 5 days after infiltration of *Agrobacterium*. Bars represent the mean + standard error (SE) from 16 infiltration sites. Statistical differences were assessed using analysis of variance (ANOVA) with *post‐hoc *Tukey testing.

Fig. S2 Alignments of BLN06, BLR38 and BLR40 with related *B. lactucae* effectors. BLAST was performed with the amino acid sequences of BLN06, BLR38 and BLR40 against the transcriptome set derived from *L. sativa* cv. Olof infected with Bl:24 material, with a cut‐off of 1e^-05^. Hits were compared with the *B. lactucae* effectorome to identify the related effectors. Alignments were created using Clustal Omega.

Fig. S3  Subcellular localization of effectors *in planta. *(a) *Bremia lactucae* effectors BLN06‐YFP, BLR38‐YFP and BLR40‐YFP were co‐expressed with free CFP in *Nicotiana benthamiana *using *Agrobacterium*‐mediated transient transformation. Infiltration sites were cut out 2 days after infiltration and incubated in a 5 mg/mL propidium iodide (PI) solution to stain the cell wall. Effectors BLN06 and BLR40 are localized at the plasma membrane, whereas BLR38 resides in the nucleus. Free CFP was used as a marker for the cytoplasm and the nucleus. Bars are 10 µm. CFP, cyan fluorescent protein; YFP, yellow fluorescent protein; (b) BLN06‐YFP and BLR38‐YFP were expressed in *L. serriola* LS102 alongside untagged versions, and BLR40‐YFP was expressed in *L. sativa *cv. Design alongside untagged BLR40. Bars represent the mean + standard error (SE) from 12–16 infiltration sites. Statistical differences were assessed using analysis of variance (ANOVA) with *post‐hoc *Tukey testing.

Fig. S4  Expression of effectors *BLN06*, *BLR38* and *BLR40* during *Bremia lactucae* infection. *Bremia lactucae* effectors *BLN06* (a), *BLR38* (b) and *BLR40* (c) are expressed during infection of *Lactuca sativa* cv. Olof with Bl:24. Seedlings were harvested at 3 h and 1, 3 and 6 days after inoculation. Data of three biological replicates are depicted as the mean + standard deviation (SD). Effector transcript levels were calculated relative to *B. lactucae* *ACTIN*. (d) *Bremia lactucae* *ACTIN* transcript levels were calculated relative to* L. sativa* *ACTIN*.

Fig. S5  Susceptibility of BLR38‐recognizing and BLR38‐non‐recognizing families to multiple *Bremia lactucae* races. Leaf disc assays were performed on BLR38‐recognizing (black bars) and BLR38‐non‐recognizing (grey bars) *Lactuca serriola* LS102 × *L. sativa* CobhamGreen F3 families with various (a–e) *B. lactucae *races (50 spores/µL). Leaf discs were scored at 11 days after inoculation. Scores: 9, fully susceptible; 7, 75% sporulation; 4, some spots with sporulation; 3, sporulation on the leaf disc edge only; 1, resistant with necrosis; 0, fully resistant. Error bars indicate standard deviation. Statistical differences were assessed using analysis of variance (ANOVA) with *post‐hoc *Tukey testing.

Fig. S6  BLR38 alleles are recognized in *Lactuca serriola *LS102. BLR38 alleles BLR38^I82M^ (a), BLR38^V188F^ (b) and BLR38^I82M V188F^ (c) with non‐synonymous single nucleotide polymorphisms (SNPs) in the effector domain are recognized in *L. serriola* LS102. Hypersensitive responses were scored 3 days after infiltration of *Agrobacterium*. Bars represent the mean + standard error (SE) from six to eight infiltration sites. Statistical differences were assessed using analysis of variance (ANOVA) with *post‐hoc *Tukey testing.

Fig. S7  Fine mapping of BLR38 responsiveness on chromosomes (chr) 4 and 8. Resistance to Bl:22 and recognition of BLR38 were fine mapped in *Lactuca serriola* LS102 × *L. sativa* GreenTowers F3 family #108 on chr 4 (a) and chr 8 (b). The previously defined smallest mapping intervals required for the recognition of BLR38 are depicted with vertical grey bars. The new smallest mapping interval is indicated with a broken grey line within the previous interval. The significance thresholds for the logarithm of the odds (LOD) scores are depicted as broken horizontal lines.

Fig. S8  Reference scoring matrix for *Agrobacterium* infiltrations in lettuce leaves. The scores range from ‘0’ (no necrotic lesions or chlorosis) to ‘4’ (severe necrotic lesions and chlorosis).

Table S1  *Bremia lactucae* effectors and amino acid sequences.

Table S2  Overview of effector responses.

Table S3  Overview of validated effector responses.

Table S4  Genes with nucleotide‐binding and leucine‐rich repeat receptor (NLR)‐associated domains in *Lactuca sativa* cv. Salinas that are located within the mapping intervals associated with BLR38 recognition.

Table S5  Primers used in this study.
