# Supplementary material for: Recognition of lettuce downy mildew effector BLR38 in Lactuca serriola LS102 requires two unlinked loci
Source: Mol Plant Pathol. 2018 Nov 6;20(2):240–53. doi: 10.1111/mpp.12751 (PMC6637914; doi:10.1111/mpp.12751)
Supplement: Supplementary file 2 — Fig. S2 Alignments of BLN06, BLR38 and BLR40 with related B. lactucae effectors. BLAST was performed with the amino acid sequences of BLN06, BLR38 and BLR40 against the transcriptome set derived from L. sativa cv. Olof infected with Bl:24 material, with a cut‐off of 1e-05. Hits were compared with the B. lactucae effectorome to identify the related effectors. Alignments were created using Clustal Omega. [file MPP-20-240-s002.pdf]

Alignment of BLN06 with BLN01

```
BLN06      MTLLHCWLLLVGHLASTAYADFITKDFKS--LPPPAYDTNATQALVPYNAALEERNGPSS
BLN01      MNL--RVLVLAVFLAATTRGDLATDDVNSTSLRLPSIEKRAD----GYGNAFNEEKV---
           *.*    *:*. .**:*: .*: *.*.:*  *  *: :..*      *. *::*.:

BLN06      STALLQYI--DHKPG-LMKKLLAGLSIRFAP---PTMKVISTPTDMLRIDKIKNNIIKSS
BLN01      -LNLRQITDPSNFPNAHKKHLVAAVSKFWVKPRIPQEDLAQQTFKVFHVERVKSNLFTSK
              * *    .: *.   *:*:*.:*  :.    *  .: .   .:::::::*.*::.*.

BLN06      QWKRWARSLLEQNSM-QNSHVIITKKMMDDLKPTNFFLVLLEASKDKNTKAVAKILEETQ
BLN01      RWIKWVERVKVAIPDRTKRDEELTRIMVEEFGLPLFFQKLHEASKNPETSETAKYFERLE
           :* :*.. :        : .  :*: *::::    **  * ****: :*. .** :*. :

BLN06      FARWCTIEHESISPMEFYDVLQLNLNEPIAYMERLPVLLRYWKY----YKSVHSPMSSVA
BLN01      YDRLL---KNKVFPEEFREQLGLQHLES------DIELTRYYLKFRSRFLMLATPVEELK
           : *      ::.: * ** : * *:  *          * **:      :  : :*:..:

BLN06      TPKD------IIDKQTVNRFGPYWKHT-GEIAELLRLDFDSDSFFNHPARNVWLDLMKTY
BLN01      VPKLLKEVMDEPTKRTIELFGEFGKEEPDMVAKMFELKFNNEREVEHPLFNIWIDFMMAY
           .**           *:*:: ** : *.  . :*:::.*.*:.:  .:**  *:*:*:* :*

BLN06      LDDTKTAEPLMIKTFQLLGNAAAKNLQNNVYSPIHFAERWIQANLQPIDVVTILGLD--I
BLN01      LDEKFVASATFLQTFRLLESSAAAGNKRSLQIKETFSRRWISSDRSLKDVAKMLQLGKNQ
           **:. .*.  :::**:** .:** . :..:     *:.***.:: .  **..:* *.

BLN06      HDSNLATNSAFSFLKVFIEKFLVNHPEADTTVVKIFSRLGSDESEKALALRKSFVSFFLR
BLN01      ADSSWINRLELQLYMSYMRKYILTHRKADPMLSWVLSKLVGLKHERSRKYQTRFVNFFR-
            **.  ..  :.:   ::.*:::.* :**  :  ::*:* . : *::   :. **.**

BLN06      TPKFTPKTVMSIFDLTISADYVEKNPVWAIWMEYVNVYLVKNKVCPEGPLADTLEFLGST
BLN01      -DNFQPSEVLTILRLDNEGGQIKNRPLVEFGLQYVASFLLKNPKAEPSI-LKTLKLLCHH
             :* *. *::*: *  ... :::.*:  : ::**  :*:**  .  .   .**::*

BLN06      AAADGVV-----------------------------------------------------
BLN01      GNSDDMFAFAKLWVSLETTPETFLKMLGIKKIDASILNHDLRDLWLAFLAHYGARLPSQA
           . :*.:.

BLN06      ----------------------RKKSIELLYSSWSGKTSQDTRIKQFLTAAARLNQLEL*
BLN01      LPHEMLQTINHLTTSAMMDNRSSREALAKIFHFWSRKNLTQDDMFKMLRLHTFRPHYFIN
                                 ::::  ::  ** *.  :  : ::*   :   :  :

BLN06      ------------------------------------------------------------
BLN01      PLLSTWDMYQLTFVTMHPTEPQQQLSDMIFRCFNVNDVEKLTAGAKHIPDMLHSKVKTVV


BLN06      ---------------
BLN01      KELQDKVDHFFGRT*
```

Fig. S2, Alignments of BLN06, BLR38 and BLR40 with related *B. lactucae* effectors. BLAST was performed with the amino acid sequences of BLN06, BLR38 and BLR40 against the transcriptome set derived from *L. sativa* cv. Olof infected with Bl:24 material, with a cut-off of 1e^{-05}. Hits were compared with the *B. lactucae* effectorome to identify the related effectors. Alignments were created using Clustal Omega.

Alignment of BLR40 with BLR19, BLR31 and BLR32

```
BLR40      MLLSRAISVVALLACICCGVHTQDSKADLGTLRT-TDSAIITSQRRLRTSVDLVDNEERF
BLR31      MLLSRAISVLALLACIRCGVHAQNTEQNLKTQLT-TDSAMITSQRLLRTSVDFKDSEER-
BLR32      MNLIRAMFVAALVACTRNGVHAKASEADLVTLLT-TNSEIVTSQRLLRTSAEPDDNDERA
BLR19      MLLSRAISVVALLACIXCGAHAQDSEVDLGTLLTTLDSSMVTSQRLLRTSVDLDNNEERV
           * * **: * **:**   *.*:: :: :* *  *  :* ::**** ****.:  :.:**

BLR40      RWPFQQFFKDRWHRKQIKTYFRDQK-----------DNVSEGLV-EQLIARHGLKNVEKV
BLR31      -WPTES----SRIRSAIKDYFREFP-----------EKVSIAMA-IRQIDAHGVRHVEKV
BLR32      I--NIPFITNYNQRTRIKAPLKELKKDSNILSNVKQRLALERVVRKEIYNRGGYQNAKKV
BLR19      KWPFQNLVTDYLNQKAIRKSLVNQAKKTVN---AHDENVLEEAV-KKEINAGRVKNVKQA
                        :. *:  : :                .    .  .       ::.::.

BLR40      LSEVKFPLAVQISIRKILVNYKGKQAFTRPHLTPADTL*-------
BLR31      LSQYKFPAADQGNIRLAIIHHKAPK*--------------------
BLR32      LEETDVNDPGRAILNSHVNLYKWFHNVDK*----------------
BLR19      LSKLKNGDPAKAKLQRLY-NAEILRNLPKTHNSGQVRISRDKVSR*
           *.: .     :  :.      :  :
```

Alignment of BLR38 with BLR17, BLR28 and BLR33

```
BLR38      -MHCTVFFLLIACAKSSYGQTRSVSTAK----SESKSDEYSYNSDAIDQSRLLRGAVNPV
BLR33      MLFSVFFFLVATCVKSSYGHSVAVSTRD-PENIALQLHEYASIPETIETIRRLRGALAHD
BLR28      -MLRVVLFLVAACAKTSYSHTVALSTRN-SQYIASKANEHATIPEDINLNRRLRKAAVIT
BLR17      -MFSTVLFLVAACAKSSYGHSVSIFTRDSTKYIASNFDEYSTIPEDIDAKRRLREAVGVD
            :  ..:**: :*.*:**.:: :: * .        : .*::   : *:  * ** *

BLR38      SENMALRKFIFDMPEMLRPEH-----------F------EPIFINPAAVKEVIKDYLAYG
BLR33      ATAYDERMFFENAANKMYAIAQKTR--LSAAAVKKLIPESQEKKLLSYYLTKMKGFIKDR
BLR28      EVAETLESIIEAF-NPLRTLRSDV---RSEMSSKTKLEQDAMLKEPSFYFRMLKPFSEFR
BLR17      GIARDAEKTFADIRHSLDRLNKDFVRSNSFKNINPLITAEALVKQPSFYQEAFLPLITAR
                 .  :    . :                         .      :     :

BLR38      EALCGSGYDPRLLALFGVRPTVLKQELMKAKGVTLSVMPSSRKRPRALDEVESEVENFRN
BLR33      EALYSITSSYDELALLGVTPDLFRTRLLSVESP---------------EVAAARAIEYEE
BLR28      IRACFEVYEIDTLILFGTSPHLLKQYIQNGIPRGIL-----PESVTVLATTGEKLKRFQR
BLR17      GIKCSKDFEFATMALLGVSPGTLRQKIQVAAQQPSN-----IWSYQTSEYNEHFVASYKK
                   .   : *:*. *  ::  :                              :..

BLR38      VLKDFFIPPTTTNPSKLIPDDIETLVPEHVSAHFN-----SLVYLMYFAVLHF-DSQELA
BLR33      YIKNICFVSSEKNPCKTVTELV---ESNKFDKIKELMEKHSIAELLIGALTNLNKVNHLD
BLR28      QFDIFFNPPTGSKPSKPSRAWPYARGPQVQANFKKI-YSSDHIKFLAYAFHHLDDVNILA
BLR17      YLDVVFMAPTISESSAFVKKHISMSIPEPSEMTYKL-LNAAVIQLLQLAIRNVDDVNELA
            :. .    : .: .            :      :          ::  *. :. . : *

BLR38      TMSSSVLLKYALQKNLLLREKIESGTLGEWERDFRLMRVLNVYKSEQT*-----
BLR33      KLTTRIQLKYVLENTPELGPLLSR-DVNELLKDPSVSKIFSRFL-TFVYGIPS*
BLR28      KLSSSIIYRFVLDNFKECRATIRYGTVEDWYKHPMLNKLLRVHEVCRKFGI*--
BLR17      KLATSVTFKYALEHDEEFSTIMMFGNLEAWISNPVLNKLLMVHQVLLKS*----
           .::: :  ::.*::       :    :     .  : :::  .
```

Fig. S2 continued.
