# Supplementary material for: Recognition of lettuce downy mildew effector BLR38 in Lactuca serriola LS102 requires two unlinked loci
Source: Mol Plant Pathol. 2018 Nov 6;20(2):240–53. doi: 10.1111/mpp.12751 (PMC6637914; doi:10.1111/mpp.12751)
Supplement: Supplementary file 8 — Fig. S8 Reference scoring matrix for Agrobacterium infiltrations in lettuce leaves. The scores range from ‘0’ (no necrotic lesions or chlorosis) to ‘4’ (severe necrotic lesions and chlorosis). [file MPP-20-240-s008.docx]

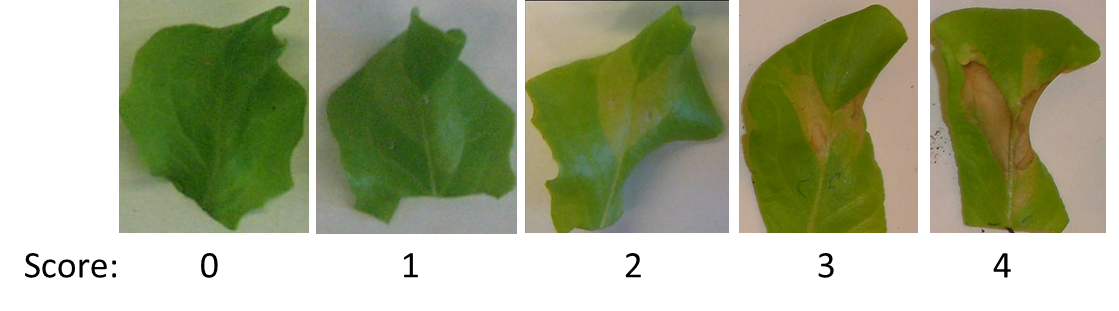


**Fig. S8,** **Reference scoring matrix for *Agrobacterium* infiltrations in lettuce leaves.** The scores range from 0 (no necrotic lesions or chlorosis) to 4 (severe necrotic lesions and chlorosis).
