# Supplementary material for: Recognition of lettuce downy mildew effector BLR38 in Lactuca serriola LS102 requires two unlinked loci
Source: Mol Plant Pathol. 2018 Nov 6;20(2):240–53. doi: 10.1111/mpp.12751 (PMC6637914; doi:10.1111/mpp.12751)
Supplement: Supplementary file 11 — Table S3 Overview of validated effector responses. [file MPP-20-240-s011.docx]

**Table S3. Overview of validated effector responses**

| *Accession/ line* | *Lactuca species* | *Resistance genes* | *Effector* | *Effector response validation* |
| --- | --- | --- | --- | --- |
|  |  |  |  |  |
| LS102 | *serriola* | *Dm17* | BLR38 | Necrotic response |
| LS102 | *serriola* | *Dm17* | BLN06 | Chlorotic response |
| NUNDM17 | *sativa* | *Dm17* | BLN06 | Necrotic response |
| RYZ2164 | *sativa* | *Dm17* | BLN06 | Chlorotic response |
| CGN05318 | *saligna* |  | BLR35 | No response |
| PI491000 | *saligna* |  | BLN05 | No response |
| CGN14263 | *serriola* | *Dm43/ Dm44* | BLR35 | No response |
| CGN14263 | *serriola* | *Dm43/ Dm44* | BLR36 | No response |
| CGN14263 | *serriola* | *Dm43/ Dm44* | BLR38 | No response |
| Design | *sativa* |  | BLR40 | Necrotic response |
| Colorado | *sativa* | *Dm18* | BLR31 | No response |
