# Supplementary material for: Recognition of lettuce downy mildew effector BLR38 in Lactuca serriola LS102 requires two unlinked loci
Source: Mol Plant Pathol. 2018 Nov 6;20(2):240–53. doi: 10.1111/mpp.12751 (PMC6637914; doi:10.1111/mpp.12751)
Supplement: Supplementary file 12 — Table S4 Genes with nucleotide‐binding and leucine‐rich repeat receptor (NLR)‐associated domains in Lactuca sativa cv. Salinas that are located within the mapping intervals associated with BLR38 recognition. [file MPP-20-240-s012.docx]

**Table S4, Genes with NLR associated domains in *L. sativa* cv. Salinas that are located within the mapping intervals associated with BLR38 recognition**

| **Locus chromosome 4*** | | |  |
| --- | --- | --- | --- |
| *Alias^‡^* | *Domains^#^* | *Protein length (aa)* |  |
| Lsa017025.1 | TIR | 333 |  |
| Lsa005833.1 | TIR, Protein tyrosine kinase | 499 |  |
| Lsa007832.3 | TIR, NB-ARC, LRR | 1302 |  |
| Lsa031407.1 | TIR | 103 |  |
| Lsa031413.1 | NB-ARC, LRR | 934 |  |
| Lsa022931.1 | LRR | 421 |  |
|  | | |  |
| **Locus chromosome 8**^†^ | | |  |
| *Alias^‡^* | *Domains^#^* | *Protein length (aa)* |  |
| Lsa013198.1 | LRR | 596 |  |
| Lsa016543.1 | LRR, Ubiquitin elongating factor core | 552 |  |
| Lsa008487.1 | LRR, Protein kinase | 624 |  |
| Lsa043030.1 | TIR, NB-ARC, LRR | 1157 |  |
| ^*^ defined as the smallest mapping interval positioned at 274,187,931 to 292,881,001bp in *L. sativa* Salinas genome version 8 | | | |
| ^†^ defined as the smallest mapping interval positioned at 47,352,309 to 55,420,000 bp in *L. sativa* Salinas genome version 8 | | | |
| *^‡^* according to the Lettuce Genome Resource [http://lgr.genomecenter.ucdavis.edu](http://lgr.genomecenter.ucdavis.edu/) | | | |
| ^#^ identified with Pfam | | | |
